# Who you gonna call? Examining police reports involving people with psychosis to improve front line management

**DOI:** 10.3389/fpsyt.2025.1728409

**Published:** 2026-02-20

**Authors:** George Karystianis, Freya Stephenson, Patricia Taflan, Sharon Reutens, Ed Heffernan, Tony Butler

**Affiliations:** 1 School of Population Health, University of New South Wales, Sydney, NSW, Australia; 2 School of Psychiatry, University of New South Wales, Sydney, NSW, Australia; 3 Mental Health Unit, Queensland Police Force, Brisbane, QLD, Australia

**Keywords:** Australia, mental health, mental health crises, police, psychosis

## Abstract

**Background:**

In New South Wales (NSW), Australia, police respond to mental health related events every nine minutes, sometimes resulting in negative outcomes, including arrest, use of force and death.

**Methods:**

In this exploratory study, we manually analyzed 100 “patient” narratives (i.e., narratives with the involvement type for the suspected individual set by police as “patient”) to describe police encounters with individuals reported to have psychotic disorders in NSW from 2017 to 2021 to identify the reasons for police involvement, arrest circumstances, and outcomes.

**Results:**

Of 100 events, 59% involved males, 40% females, and one transgender patient. Most (60%) reported multiple mental illnesses, and 44% involved substance use and alcohol. Half (53%) occurred at the patient’s residence, followed by public places (17%). Police were called by the patient (25%), or a family member/partner (24%). Common reasons included welfare concerns (22%) and domestic disputes (18%). Only 11% resulted in arrest, while 71% resulted in transport to hospital/mental health unit. More than half (55%) resulted in the patient being scheduled under the Mental Health Act. Use of force was minimal, reported in 5% of events.

**Conclusions:**

Our preliminary findings challenge the stereotype of “dangerous” mentally ill individuals, with most police encounters involving welfare outcomes. Interactions with police such as those examined here present an opportunity for mental health services diversion, and cross-sectoral collaboration to address community care gaps. This study underscores the role police play in managing those experiencing mental health crises and the need for police training to effectively manage these situations.

## Introduction

Police and law enforcement officers often engage with individuals experiencing mental health crises (1–5). It is estimated that mental health-related calls represent a substantial proportion of police emergency responses (4), with approximately one in ten individuals with mental illness encountering police en route to care (6, 7). Indeed, in NSW it is estimated that police are called to events involving mental health every nine minutes (8).

In Australia, police are legally required to provide 24/7 responses to community mental health calls (9). However, police officers mostly respond unassisted by service providers such as doctors, paramedics, social workers and other health care professionals (10). Estimates show that Australian police spend 10% to 30% of their time managing mental health cases, including locating individuals who have left psychiatric care, connecting people to services, and addressing self-harm or suicide situations (11, 12). While such involvement is often necessary and can be beneficial, outcomes during these encounters can be negative. For example, 85% of fatal police shootings of individuals with mental illness in Australia involved failure to follow police directives and possession of a weapon (13).

As these encounters with police are frequent (14), the arresting or contact circumstances vary, including both non-criminal and criminal incidents such as street stops, domestic disputes, suspicion of criminal offences, public disturbances, verbal or physical altercations, reporting a crime, and seeking help, often in the context of a mental health crisis (1, 3, 15, 16). Police officers typically respond to low-level misdemeanor offences and calls involving people who have mental illnesses rather than to violent events (17). Arrest rates of such individuals appear more likely to be influenced by the behavioral manifestations of mental illness than by the presence of mental illness itself (18). Research shows that higher arrest rates in this group are due to police responses to an individual’s presenting behavior which can be perceived as disrespectful or hostile (18). A German study found out that most of 1,000 police officers experienced interactions with persons with mental illness as conflictual due to behaviors they perceived as unpredictable and irrational (3).

Without adequate mental health training and with a mandate to preserve public safety, increased police interaction in mental health crises can be conflictual, leading to injuries and fatalities (3, 19–21). Officers are expected to identify mental illness related behaviors despite lacking clinical training (3, 22, 23). Assessing potential risks and behaviors of individuals is challenging for the officers involved (5, 24) due to verbal and physical aggression, communication difficulties, and insufficient knowledge about mental health, which contribute to the onset of anxiety for over one quarter of officers (3). Additionally, police officers tend to approach people with mental illnesses as high-risk individuals which can result in a self-fulfilling prophecy (25, 26). Research shows those with certain mental health conditions are more likely to threaten or use weapons against officers (27).

Such concerns have also been raised by people with mental illness about police interventions, particularly those involving the use of force (28) raising the likelihood of police violence (5, 29) and fatal outcomes (30, 31) and posttraumatic stress for police officers (32). Laniyonu and Goff (33) found that experiencing police use of force (and injury resulting from it) is about 11 times higher for people with serious mental illness than for people without a mental illness. For example, in Canada, between 23% and 70% of fatalities during police encounters were related to mental health or substance use concerns (21). In the US, 40% of individuals injured as a result of police force suffer from mental illness (30). Additional studies in the United Kingdom have found that 37-48% of individuals fatally shot by police were classified as having a mental health problem at the time of the shooting (34, 35). In Australia, Randall et al. (36) conducted interviews with participants who were apprehended by police while experiencing a mental health crisis or due to their erratic behavior in public spaces (e.g., walking along highways). Although portrayals of police interactions with people with mental illness are often overly negative, research has shown that almost 75% of individuals with mental illness view their law enforcement contacts positively, despite experiencing physical restraint (37).

Internationally, police forces employ different interventions to prepare for these encounters, ranging from street triage (police and health care joint response) to crisis intervention teams (CIT), communication training and the use of tasers (2, 7, 38–40). Street triage programs have been effective in decreasing decrease traumatic experiences and stigmatization of individuals with mental illnesses (40), while CIT programs can reduce the incidence of violence used by police against people with mental illnesses and the impact of negative stereotypes for this group (41, 42). Studies have looked at several resource-intensive aspects of police dispatches during mental health crises (1, 43), but there is still insufficient knowledge surrounding the reasons for police dispatches.

Explanations for the occurrence of these interactions are complex, but generally focus on clinical risk factors, such as co-occurring substance use problems and non-adherence to treatment, as well as social and systemic factors, including deinstitutionalization policies, homelessness, poverty, community disorganization, poorly funded and fragmented community-based services, hospital emergency room bed pressures, overly restrictive civil commitment criteria, intolerance of social disorder, and criminal law reforms (44, 45). However, a review of police training programs, which focused on changing attitudes and behaviors (46) demonstrated that the effectiveness of these programs was questionable in large part due to the relatively poor quality of the research designs used to evaluate them. It was also noted that there are significant differences in existing training programs across Canada, the United States, United Kingdom, and Australia (46).

Police estimate that among individuals they encounter, those with mental disorders, mood and substance use problems are the most prevalent (24, 27). Individuals with psychotic disorders (e.g., schizophrenia) have the highest risk for violent offences and fatal outcomes (27, 47) and thus, are overrepresented in cases in which the police use force (27) with higher arrest rates than in community samples without mental illness (48, 49). Reasons behind the arrest of people with schizophrenia may be due to the behavioral manifestations of the disorder (50) and the associated psychosocial adversity (51). However, for the same reasons, such individuals can also be a potentially vulnerable population at risk of victimization in the community (52, 53).

Little is known about how police officers’ conduct is perceived and what kind of behaviors are recommended during encounters with individuals who suffer from psychotic disorders. While Australian studies and reports often indicate that family members play crucial roles in initiating police contact during mental health crises (54), there are few studies that break down how police come into contact with people experiencing a mental health crisis (1, 3, 27, 55). This includes who called the police, the reason police were called, and the location of police attendance. Understanding these factors offers important insights into how we can rethink police responses to those experiencing mental health crises. This exploratory study seeks to identify the characteristics of police interventions (i.e., premises type where intervention occurred, illicit drug use, reason for police involvement) involving individuals with a reported mention of psychotic disorder (e.g., schizophrenia, psychosis) in the NSW Police Force (NSWPF) records from 2017 to 2021, where the involvement type of an individual was listed as “patient”, to gain a deeper understanding of the dynamics between police and individuals with mental illness, including escalating behaviors of both the attending police officers and the mentally ill individuals.

## Methods

### Data

Police records in the NSWPF comprise structured data (called fixed fields) and text narratives. The fixed fields contain demographic information such as the sex, date of birth, postcode, type of premises, on individuals – victims, and persons of interest (POI), individuals suspected or charged with a crime - involved in a variety of event types (e.g., domestic violence, theft), and any charged offence(s). The text narratives consist of unstructured text describing the circumstances of the police event, including its cause, persons present, any substance and/or alcohol use, the mental health status of POIs and victims, victim injuries, types of abuse, property damage and any actions taken by police, among other details. To examine events involving people with psychotic disorders, we obtained 203,062 NSWPF records from 2017 to 2021 that were flagged as “patient”. Such events involved individuals who had come to the attention of police due to a potential mental health problem, and thus recorded as “patients” rather than “victim”, “witness” or “POI”. The individuals recorded with mental illness in these events are hereafter referred to as “patients”. Narratives were made available by the NSWPF from their COPS (Computerized Operational Policing System) system as part of a Medical Research Future Fund (MRFF) study (MRF2005635) to examine the mental health needs of populations during pandemics. A formal data sharing agreement was developed, and ethics approval granted by the University of New South Wales (HC16558).

To identify mentions of psychotic disorders (e.g., psychosis, schizophrenia) in the narratives, we applied a text mining method devised by the first author (56). Briefly this method involved the design of rule-based language expression patterns combined with dictionary terms for the recognition of mental health mentions through General Architecture for Text Engineering (GATE) (57). The methodology has been published elsewhere and has been evaluated on police narratives. From the returned results (15,247 mentions of a psychotic disorder), we randomly selected 100 narratives (20 per year) using a Python script. We examined factors related to the encounter between police and individuals with mental illness. Annotation guidelines were created by the first and fourth authors with expertise in analyzing police narratives and psychiatry respectively to identify:

- sex of patient
- substance use/abuse (including alcohol and medications)
- mental illness mentions
- caller relationship to patient
- reason for police intervention
- arrest status
- event location
- patient behavior during police interaction.

The sample was manually annotated by the first and last authors. No difference in annotations was observed across all selected features. The identified mental illness mentions ranged from general descriptions (e.g., depressive disorder, mental health issues) to specific illnesses (e.g., post-traumatic stress disorder, attention deficit hyperactivity disorder) and these were mapped to the World Health Organization’s International Classification of the Diseases (ICD-11) Mental and Behavioral Disorders (58) across five category levels by the fourth author (SR) with expertise in psychiatry.

Multiple reasons and locations can be detailed in a single narrative. We decided to use the initial reason that police was involved and the location the police first encountered the patient.

### Ethics

Permission to access the narratives was granted by the NSWPF following ethics approval from the University of New South Wales Human Research Ethics Committee (reference: HC16558).

## Results

### Demographics, mental health and substance use

Of the 100 events, 59% involved males, 40% females, and one transgender patient. All events mentioned mental illness, with 60% reporting unspecified psychosis, 38% a psychotic disorder induced by an unknown or unspecified psychoactive substance, and 20% schizophrenia. A total of 15 different mental health conditions were reported. These included mood disorders (depression, 18% of events; bipolar disorder, 12%) followed by personality disorders (paranoid, 9%) and anxiety disorders (post traumatic stress disorder, 7%). A total of 38% of events had one mental illness, while 31% had two, 19% had three and 12% had more than four (**Table 1**). **Table 1** in the **Appendix** lists all reported mental illnesses classified across five ICD-11 levels.

**Table 1:** Number of mental illnesses mentioned for individuals suspected of having schizophrenia or other primary psychotic disorder.

Number of mental illnesses	% of events
1	38
2	31
3	19
4+	12

Almost half of the events (44%) recorded substance use (including alcohol) with 56 mentions indicating polysubstance use (i.e., a narrative can have more than one distinct illicit drug mention). Methamphetamine (i.e., ice) (18%) was the most common, followed by cannabis (13%). Only six events reported that the patient was under the influence of alcohol during their contact with the police. Prescribed medications including antidepressants (3%), antipsychotics (11%), and anxiolytics (3%) appeared in 46% of events. At the time of encounter, 27% events mentioned that patients were taking their medication while 19% reported patients had stopped.

### Circumstances of police encounter

A total of 12 locations were identified across the 100 events. Half (53%) of the events occurred in the patient’s residence, followed by 17% in public spaces (e.g., street, park, shopping mall) and 7% at a 3^rd^ person’s residence. A further 7% of events occurred at the police station, while 5% occurred at private enterprises (e.g., motel, restaurant). The remaining 11% took place at other locations (**Table 2**, **Appendix**).

A quarter of events involved the patient calling the police (25%). Almost a quarter of events (24%) involved immediate (e.g., the parents, siblings or children of the patient) and extended family members (e.g., cousin, uncle) calling the police (19%), the emergency services (3%) or both (2%). Just over one tenth of the events (11%) included intimate partners calling the police (8%) or paramedics (3%). Other individuals who called the police included neighbors (11%), members of the public (8%) and friends of the patient (4%) (**Table 3**, **Appendix**).

The most common reason for involving the police was concern for the patient’s welfare, safety and/or mental state (22%), followed by domestic disputes involving the individual (18%). A total of 13% of events involved suicide and self-harm attempts, 12% described patients self-reporting due to paranoia or delusions, and 11% were due to disorderly public conduct (**Table 2**). A total of 16% of the events had miscellaneous reasons (e.g., intention to harm another person, 2%; attempted theft, 2%; car crash, 1%; property damage, 1%) (**Table 4**, **Appendix**).

**Table 2:** Reasons for police involvement with individuals suspected of having schizophrenia or other primary psychotic disorder.

Reason for police involvement	% of events
Concern for patient’s welfare, safety and/or mental state	22
Domestic dispute and/or domestic violence	18
Miscellaneous (e.g., attempted theft, car accident)	16
Suicide, self-harm or suicidal ideation	13
Paranoia and/or delusions	12
Disorderly public conduct and public disturbance	11
Absconded from an involuntary hold at the mental health unit	4
Missing person or run away	4

### Police outcomes

In the majority of events (71%), police did not use any apprehension techniques. Almost one quarter of events (24%) involved more than one method of apprehension, including being searched by police (13%), restrained (9%), sedated by attending paramedics (8%), handcuffed (7%), wrestled to the ground (4%) and pepper sprayed (1%).

Half of the events (55%) concluded with the patient being scheduled under the Mental Health Act (i.e., the process of detaining someone in a mental health facility for assessment and treatment against their will). Over a third of events (36%) did not result in a schedule with 12% reporting the voluntary admission of the patient to the hospital, 13% having insufficient grounds for scheduling (i.e., patient made no threats to harm self or others), 6% seeing the patient already seeking mental health treatment, 3% lacking the reason, and 2% having the patient either calming down or refusing to talk to the police. Of the remaining 9% of events, 4% did not specify whether the patient was scheduled or not despite being taken to the hospital for assessment and 5% reported that scheduling was not applicable (e.g., the patient was deceased/non-responsive on police arrival or could not be located). Overall, almost 4 in 5 events (71%) reported the patient being taken to hospital for mental health treatment. Only 11% of the events resulted in the arrest and formal charge of the patient (7% of these were still taken to the hospital for mental health treatment).

The most common transport for mental health assessment was via ambulance with a police escort (35%) followed by unescorted ambulance (14%), police transport (12%), and driven by the patient’s family member (1%). There were an additional 9% of events where the patient was transported by an ambulance; however, these were unclear on the use of police escort.

## Discussion

We examined the reasons for police contact in 100 events involving patients with a psychotic disorder in this exploratory, retrospective study of NSW police narratives, where individuals were listed as “patients” due to suspected mental health issues by the attending police officers. The data was not analyzed according to any theoretical framework but used to report the reasons for contact by patients with the police.

### Sex and mental health

The sex distribution (males 59%, females 40%) highlighted that police interactions with individuals affected by schizophrenia or psychosis are not heavily skewed by sex. This is in line with other studies from Canada indicating that males and females with a serious mental illness had similar rates of police contact (59, 60). In our study, 62% of the events reported patients with more than one mental illness with a total of 15 different conditions. This shows the complexity of police interactions with people affected by psychotic disorders including co-occurring disorders such as alcohol and/or substance use or medication non-adherence. Depression (18%) and bipolar disorder (12%) were the most common co-existing disorders, and almost half of the events (44%) involved alcohol or illicit drugs (i.e., methylamphetamine, cannabis), or prescribed medications. Co-occurring substance use, and psychotic disorder is a recognized phenomenon (61), with the 2010 National Survey of High Impact Psychosis reporting over half of people living with psychotic illness (55%) having a lifetime history of harmful illicit drug use (62). Of the 44% of the events mentioning substance use, only 6% specifically mentioned alcohol. Although 44% of events noted substance use, only 6% mentioned alcohol, despite its 2–3 times higher prevalence in this population (63). This underreporting does not rule out alcohol use during these encounters.

### Arresting circumstances

Half (53%) of the police encounters occurred in the patient’s place of residence, followed by 17% in public spaces and 7% at a third person’s residence. This supports the prevailing concerns raised in the introduction about police interaction with mentally ill people in the community indicating unmet mental health needs (64, 65). There is evidence that community mental health services often fail to meet the needs of people with mental illness potentially increasing risks of violent encounters for individuals, the community, and service providers (66). A system is needed to protect police officers from these encounters, which can turn out violent, and to provide such individuals with a long-term health care plan. As many individuals with mental illness face or are homeless, unsupported by the existing social structure, they are likely to have increased encounters with the police (67). For an effective response to mental illness, assessments should be shared between criminal justice and mental health agencies, establishing ongoing collaboration (68).

A quarter of events involved patients calling the police or attending the police station (25%), while in two out of five events (39%), someone close to the patient (e.g., family member, partner, friend) initiated contact. Since family members, friends and intimate partners are the closest relationships, it is to be expected that they will be the first to call the relevant authorities. These findings align with existing research that explored who calls the police relating to people with mental illness (55, 69). Shore and Lavoie (69) noted that family members, patients or their friends will often contact police due to limited mental health services. Pope et al. (70) indicated, that although non-police responses are preferred, they face delay and limited availability.

Police officers respond to criminal acts - from public disturbances to violent events (71). Interestingly, in our sample, the most common reason was concern for the individual’s welfare, safe, and/or mental state (22%) followed by a domestic dispute (18%). This aligns with Wood et al. (72) who reported that most police calls involved disruptive behaviors with neighbors, or within families who felt incapable of handling a loved one with mental illness, especially when it comes to medication adherence, a common topic noted by police officers. More publicized reasons for police contact such as self-harm/suicide attempts (13%) feelings of paranoia, delusions (11%), and dangerous behavior (8%) (i.e., threatening to harm others, attempting to commit theft or property damage) (72) have been the subject of discussion, yet they were not prevalent in our data. This challenges the stereotype of mentally ill individuals as dangerous suggesting instead opportunities for treatment referral and support (36, 55).

### Police outcomes

Much attention is given to outcomes of police encounters with mentally ill people. Prior research has indicated that people with serious mental illness such as psychosis are more likely to experience force when interacting with police than people without serious mental illness (27, 33). Kesic et al. (27) found that police were 1.4 times as likely to threaten or use a weapon against people with psychotic disorder. In addition, Watson and Wood (73) reported that during mental health crises, hospital transports were more frequent than arrests. In our sample, almost one quarter of events (24%) involved techniques to apprehend (but not necessarily to arrest) patients. In some events, various apprehension techniques were used concurrently (e.g., the patient was taken to the ground, restrained and given a sedative). The use of restraints (e.g., handcuffs) was reported in less than 10% of the events while ground wrestling and the use of pepper spray were noted in only 5% of the events. As such, most events did not use force when dealing with patients. Interestingly, the fact that only a small number of patients (11%) were arrested following police interaction suggests this group is not a danger to the community. Previous research reported how people with suspected mental conditions were placed under arrest only in severe circumstances that involved a mental health crisis (15). This finding aligns with existing literature that reported that minor offences involving individuals with mental illness were more likely to result in arrests compared to those involving individuals without mental illness (1, 74). In addition, it is worth noting the possibility that attendance by uniformed police may have heightened the perception of threat among those experiencing psychotic symptoms. Conversely, it could also deter escalation into violence by establishing clear boundaries and rapid intervention, potentially averting harm to the individual, family, or bystanders (75, 76).

The most important aspect of our exploratory study was that most of these encounters (71%) involved the police transporting individuals to mental health units and hospitals. Out of 71 cases, 55% resulted in the individual being scheduled with ambulance and police escort being the most common (35%) option for transportation to hospital. In NSW, police can transfer a patient for a mental health assessment under Section 22 of the Mental Health Act as well as assist in the patient’s transfer if requested, for example, by paramedics. However, if police are considering charging the patient, they await the outcome of the assessment. Previous US research reported that service providers frequently enlist the police to transport a person for psychiatric assessment due to their unpredictable behavior and the paramedic’s inability to deal with them if a violent episode occurs (72, 73). These preliminary insights reveal how police play a crucial role in managing mental health crises, usually unassisted by professionals in this area.

### Implications for future research

While most police interactions with patients did not involve violence that does not mean the current implemented system for dealing with mental health crises in NSW is optimal. People who experience mental illness are more resistive to police commands, increasing the risk of police force (27). Training police officers to better manage mental health crises is important (2), including recognizing mental disorders (3). Therefore, such events underline the importance of fostering a partnership between the police and the mental health care system in day-to-day police routine practices. A study looking at Canadian law-enforcement organizations found that entry-level training on mental illness occurs widely, providing a strong groundwork for positive interactions (65, 77). In addition, research findings in this area have highlighted the need for high-quality studies and policies to facilitate the implementation and evaluation of approaches not involving police involvement (4). Although in NSW, the NSWPF has a Mental Health Intervention Team (MHIT) to ensure that during mental health crisis the clinical and safety needs of the person and the safety of staff and others are met in the best way possible, there is a necessity for cross-sectorial collaboration and service user input to further inform, develop, trial, and implement effective models of crisis intervention (4, 78). One such approach that could serve as a cross-sectorial blueprint is the PACER (Police, Ambulance, Clinical, Early, Response) program that embeds mental health clinicians with first responder teams to optimize outcomes for people experiencing mental health crises (79). Currently, there are models being trialed to better manage mental health crises such as the UK’s Right Care, Right Person but at this stage, they lack robust evaluation data (80).

### Limitations

Our study has several limitations. First, mentions of mental illness within the “patient” narratives are not verified by trained mental health professionals. Police officers are not trained to diagnose psychotic disorders. While attending police officers might perceive an episode of psychosis, this may not be the case. We also do not know whether patients had positive experiences with police. The narratives lack information on patients experiencing distress, anxiety or discomfort unless it is explicitly written by the attending police officer(s). Therefore, it is not possible to know whether the interaction with the police exacerbated the mental health condition of the patient. Furthermore, the lack of information on co-morbid mental health conditions or substance use does not mean they were not present at the time of the police intervention. The selected sample of narratives, featured people who were recorded by police as “patients” so any suggestions in regards the arresting circumstances and possible related factors should be taken with caution. As the NSWPF attends a mental health event every nine minutes on average (8), examination of a larger sample of events is needed to verify our preliminary findings.

## Conclusion

This exploratory study of a small sample of NSWPF narratives involving individuals (i.e., patients) suspected of having a psychotic disorder provides preliminary insights into police interactions in community settings. Our findings reveal that these encounters predominantly occur at private residences and public spaces, often initiated by patients, family members and intimate partners, with the primary reasons being concerns for welfare and domestic disputes rather than criminal acts that threatened the public. Contrary to stereotypes portraying individuals with mental illness as “dangerous”, this research showed that most interactions involved minor or non-criminal behaviors that led to hospital transport for mental health assessment. The frequent co-occurrence of substance use, and multiple mental health conditions underscore the complexity of these encounters, highlighting the need for police training to manage mental health crises and collaboration with mental health services. The study emphasizes the need for diversion programs to connect individuals into treatment and support pathways to reduce risks for individuals, communities, and police. Despite limitations, such as the small sample size and reliance on unverified police-reported mental health mentions, these findings suggest the need for evidence-based, cross-sectoral approaches to mental health crisis interventions.

## Data Availability

The original contributions presented in the study are included in the article/**Supplementary Material**. Further inquiries can be directed to the corresponding author.
