# Supplementary material for: Who you gonna call? Examining police reports involving people with psychosis to improve front line management
Source: Front Psychiatry. 2026 Feb 20;16:1728409. doi: 10.3389/fpsyt.2025.1728409 (PMC12964203; doi:10.3389/fpsyt.2025.1728409)
Supplement: Supplementary file 1 [file DataSheet1.docx]

Supplementary Material

**Table 1.** Recorded mental illnesses in 100 police narratives involving people with suspected schizophrenia or other primary psychotic disorders (World Health Organization 2025).

| **ICD-11 level 1** | **Level 2** | **Level 3** | **Level 4** | **Level 5** | **Number of events** |
| --- | --- | --- | --- | --- | --- |
| 06 mental, behavioural or neurodevelopmental disorders | Schizophrenia or other primary psychotic disorders | 6A2Z Schizophrenia or other primary psychotic disorders, unspecified | Unspecified nonorganic psychosis | Unspecified psychosis | 60 |
|  | Schizophrenia or other primary psychotic disorders | 6A2Z Schizophrenia or other primary psychotic disorders, unspecified | Unspecified nonorganic psychosis | - | 38 |
|  | Schizophrenia or other primary psychotic disorders | 6A20 Schizophrenia | - | - | 20 |
|  | Mood disorders | Depressive disorders | - | - | 18 |
|  | Mood disorders | Bipolar or related disorders | 6A6Z Bipolar or related disorders, unspecified | - | 12 |
|  | Personality disorders and related traits | 6d10 Personality disorder | 6d10.z Personality disorder, severity unspecified | Paranoid personality disorder | 9 |
|  | Disorders specifically associated with stress | 6b40 Post traumatic stress disorder | - | - | 7 |
|  | Neurodevelopmental disorders | 6A05 ADHD | 6A05 ADHD, predominantly inattentive presentation | 06 Mental, behavioural or neurodevelopmental disorders | 4 |
|  | Personality disorder and related traits | 6d10 Personality disorder | 6d10.z Personality disorder, severity unspecified | Personality disorder, severity unspecified | 3 |
|  | Neurocognitive disorders | Dementia | - | - | 2 |
|  | Neurodevelopmental disorders | 6A00 Disorders of intellectual development | 6A00.0 Disorder of intellectual development, mild | - | 2 |
|  | Neurodevelopmental disorders | 6a02 Autism spectrum disorder | - | - | 2 |
|  | Disorders due to substance use or addictive behaviours | Disorders due to substance use | 6C4Z Disorders due to substance use, unspecified | - | 1 |
|  | Neurodevelopmental disorders | 6A03 Developmental learning disorder | 6A03.Z Developmental learning disorder, unspecified | - | 1 |
|  | 6E8Z Mental, behavioural or neurodevelopmental disorders, unspecified | Mania | - | - | 1 |
|  | Personality disorder and related traits | 6d10 Personality disorder | 6d10.z Personality disorder, severity unspecified | Personality disorder, severity unspecified | 1 |
|  | Disruptive behaviour or dissocial disorders | 6C90 Oppositional defiant disorder | 6C90.0 Oppositional defiant disorder with chronic irritability-anger | 6C90.0Z Oppositional defiant disorder with chronic irritability-anger, unspecified | 1 |
|  | | | | | |
| 21 Symptoms, signs or clinical findings, not elsewhere classified | Mental or behavioural symptoms, signs or clinical findings | MB24 Symptoms or signs involving mood or affect | MB24.3 Anxiety | - | 9 |
|  |  | MB26 Symptoms or signs involving content of thought | MB26.0 Delusion | - | 9 |
|  |  | mb27 Symptoms or signs involving perceptual disturbance | mb27.2 Hallucinations | - | 2 |
|  |  | MB26 Symptoms or signs involving content of thought | MB26.A Suicidal ideation | - | 1 |
|  | Mental or behavioural disorders associated with pregnancy, childbirth or the puerperium, without psychotic symptoms | 6e20 Mental or behavioural disorders associated with pregnancy, childbirth or the puerperium, without psychotic symptoms | - | - | 1 |
|  | | | | | |
| 08 Diseases of the nervous system | Disorders with neurocognitive impairment as a major feature | 8A20 Alzheimer disease | - | - | 1 |

**Table 2.** Recorded premises type in 100 police narratives involving people with suspected psychotic disorders.

| **Premises type** | **% of events** |
| --- | --- |
| Patient’s place of residence | 53 |
| Public place (e.g., street, park, car park, shopping mall, train station) | 17 |
| Family/Partner/Friends house | 7 |
| Police station | 7 |
| Private enterprise (e.g., motel, hotel, restaurant) | 5 |
| Hospital/MH/Medical Centre | 5 |
| Police had no physical contact with patient | 2 |
| Retirement Village | 1 |
| Refugee Accommodation | 1 |
| Community Group Home | 1 |
| Bushland | 1 |

**Table 3.** Relationship of the caller to the patient in 100 police narratives involving people with suspected psychotic disorders.

| **Relationship to patient** | **% of events** |
| --- | --- |
| Patient themselves | 25 |
| Immediate/extended family | 24 |
| Mental health unit/hospital | 17 |
| Neighbor | 11 |
| Intimate partner | 11 |
| Member of public | 8 |
| Friend of patient | 4 |
| Staff at public/private premises (i.e., hotel, refugee center, restaurant) | 4 |
| Police patrol | 2 |
| Police attendance in relation to another matter | 2 |
| Landlord | 1 |

**Table 4.** Reasons for police involvement in 100 police narratives involving people with suspected psychotic disorders.

| **Reason for police involvement** | **% of events** |
| --- | --- |
| Concern for patient’s welfare, safety and/or mental state | 22 |
| Domestic dispute and/or domestic violence | 18 |
| Suicide, self-harm or suicidal ideation | 13 |
| Paranoia and/or delusions | 12 |
| Disorderly public conduct and public disturbance | 11 |
| Absconded from an involuntary hold at the mental health unit | 4 |
| Missing person or run away | 4 |
| Assistance for scheduling | 3 |
| Intention to harm another person | 2 |
| Breach of Apprehended Domestic Violence Order | 2 |
| Attempted theft | 2 |
| Physical altercation and/or verbal argument (non-domestic) | 1 |
| Car crash | 1 |
| Property damage | 1 |
| Patient alleges to have been sexually assaulted | 1 |
| Odour emitting from house | 1 |
| Fell out of window | 1 |
| Concern for child in care of patient | 1 |
